# Augmented Articulating Spacers in Infected Total Knee Arthroplasty: Surgical Technique

**DOI:** 10.3390/healthcare12070735

**Published:** 2024-03-28

**Authors:** Domenico De Mauro, Enrico Festa, Donato Di Gennaro, Tiziana Ascione, Giannantonio Coletta, Massimo Mariconda, Giovanni Balato

**Affiliations:** 1 Department of Public Health, Orthopedic Unit, “Federico II” University, Via S. Pansini, 5, 80131 Naples, Italy; 2 Department of Orthopedics and Geriatric Sciences, Catholic University of the Sacred Heart, 00168 Rome, Italy; 3 Department of Orthopedics and Rheumatological Sciences, Fondazione Policlinico Universitario A. Gemelli IRCCS, 00168 Rome, Italy; 4 Service of Infectious Diseases, AORN A. Cardarelli Hospital, 80131 Naples, Italy

**Keywords:** periprosthetic joint infections, total knee arthroplasty, two-stage, 1.5-stage, articulating spacer, metal-on-poly

## Abstract

Periprosthetic joint infections (PJIs) are a prominent subject of discussion in orthopedics and are frequently debated at conferences and congresses. In the context of PJIs affecting the knee, the decision between following a one-stage or two-stage treatment approach has historically been a pivotal consideration. The first option is limited by indications and potentially devastating complications in case of failure, whereas the second is widely accepted as the gold standard. Initially, the spacer was conceived solely to restore and maintain knee space after removal of the implant. An articulating spacer was introduced to mitigate patient limitations and improve knee function and quality of life. Two main types of articulating spacers are utilized in knee PJI treatment: the mold spacer and the metal-on-poly spacer. This text outlines a technique for metal-on-poly spacer implants. Based on our experience and the existing literature, this approach facilitates early full weight bearing and faster recovery of the knee’s range of motion, ultimately improving the quality of life after surgery, thus allowing the spacer retention for an extended period, as suggested by the 1.5-stage revision.

## 1. Introduction

Periprosthetic joint infections (PJIs) are a prominent subject of discussion in orthopedics, frequently debated in conferences and congresses [1,2]. Their growing significance in daily orthopedic practice globally, coupled with the intricacies of diagnostic and treatment algorithms, contributes to the heightened interest in this area [3].

In the context of PJIs affecting the knee, the decision between following a one-stage or two-stage treatment approach has historically been a pivotal consideration. The first option, prevalent in Europe but on a smaller scale than the two-stage approach [4], is limited by indications and the potentially devastating complications in case of failure [5]. The two-stage approach, on the other hand, is widely accepted as the gold standard for treating PJI globally, with various variations in its implementation [6].

The two-stage technique involves a two-surgery approach, with the second surgery delayed based on factors such as the antibiotic regimen, blood and synovial tests, and soft tissue conditions [7]. Initially, the spacer was conceived solely as a means to restore and maintain knee space after the removal of the implant. It was essentially a mass of antibiotic-loaded bone cement, occasionally reinforced with metal bars, analogous to the use of reinforced concrete in construction. While this technique is still commonly employed, it results in a temporary arthrodesis, significantly impacting the quality of life and knee function in patients. According to the literature, its use is largely suggested in bad soft tissues, extensor mechanism rupture, and huge bone loss [8]. 

To mitigate patient limitations and improve knee function and quality of life, the concept of an articulating spacer was introduced [9]. Two main types of articulating spacers are utilized in knee PJI treatment: the mold spacer and the metal-on-poly spacer. The mold spacer is made solely from bone cement, available in limited sizes, and preformed to resemble the appropriate femoral and tibial prosthetic components [10]. Advantages of this kind of spacer are limited costs, ease of crafting, and easier removal in the second stage. The limitations of this spacer type include a limited range of sizes in most cases, the inability to address asymmetrical deformities, lower mechanical resistance, and moreover, lower ROM and knee function after the first stage in two-stage revision [9].

The metal-on-poly spacer, instead, is a technique first proposed by Hofmann et al. [11]. Originally, this technique involved the refurbishment of the existing femoral component through meticulous cleaning and sterilization [12]. However, contemporary practice has evolved to incorporate a new femoral component, coupled with a fresh polyethylene liner directly cemented onto the tibia. Notably, studies have demonstrated that the use of a metal femoral component does not correlate with an increased risk of infections or spacer failure attributable to infection [9]. This design facilitates earlier mobilization, enabling patients to utilize the spacer as a primary knee. However, the pitfall of this technique is the inability to adequately address significant bone defects and articular gaps, leading in some cases to an unstable and painful knee in those patients treated with this type of spacer, significantly impacting their quality of life during the interim period before reimplantation [13]. 

A more stable articulating spacer can be incredibly beneficial, especially in the context of the 1.5-stage revision technique. This approach is increasingly utilized worldwide, involving the lifelong retention of the articulating spacer in select patients who cannot undergo revision due to infection or specific patient characteristics. This reflection prompts a significant reconsideration of articulating spacers and the associated surgical techniques. The goal is to offer patients a balanced solution that addresses the imperative to combat infections while ensuring a good quality of life and optimal knee function [14].

## 2. Indications and Patients’ Selection

Candidates for the articulating spacer are those patients presenting with small bone defects, competent collateral ligaments, and intact extensor mechanism, without severe soft tissue compromise. Otherwise, this technique is contraindicated in patients with a history of multiple articulating spacers, who are generally deemed unsuitable candidates for the balanced articulating spacer approach. Conversely, the 1.5-stage strategy serves as a valuable alternative treatment option, especially for individuals who are unable to undergo a two-stage operation due to underlying morbid conditions or financial constraints. Additionally, it proves beneficial for patients who express satisfaction with the temporary spacer solution.

Residual bone loss assessment can be meticulously conducted during preoperative planning using X-rays or, in cases requiring further clarity, CT imaging may be utilized. Intraoperatively, common measurement devices available in total knee arthroplasty (TKA) instrumentations enable the identification and management of potential defects. While preoperative planning aids in the approximate prediction of augment and cone sizes, intraoperative measurements are essential for confirming or suggesting the correct sizing required for knee balancing.

## 3. Surgical Technique 

### 3.1. Step 1: Positioning and Surgical Approach

The patient is positioned supine on the surgical table for the procedure. Two cylindrical supports are placed at the distal end of the table to allow both 90° flexion and hyperflexion of the knee during the surgery. A previous skin incision is generally used as a surgical approach. In the presence of different scars, we used the most lateral to prevent devascularization of the lateral skin flap [15]. A new incision is used if a bridge of at least 6–7 cm between scars is present.

### 3.2. Step 2: Deep Surgical Debridement and Synovectomy

**Figure 1:**
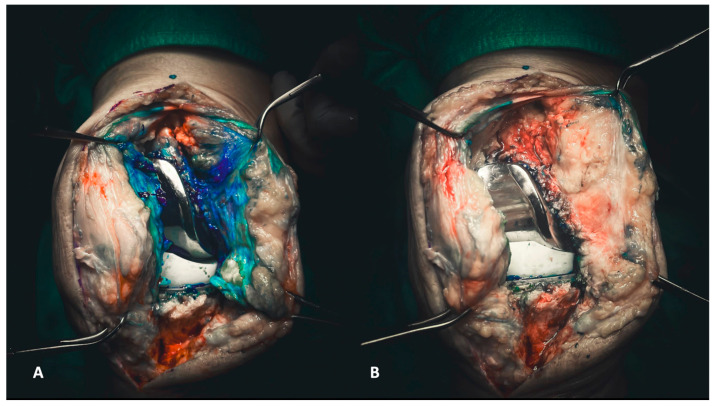
(**A**,**B**) Injecting methylene blue into the joint enables the precise delineation of targeted tissues for subsequent debridement (**A**). Consequently, the debridement process becomes more discernible, allowing the surgeon to visually assess the entirety of the debridement directly (**B**).

Before initiating surgical access to the joint, obtaining a synovial fluid sample is crucial for laboratory examinations. Following arthrocentesis, the same needle injects diluted methylene blue into the joint, as previously described [16]. This step enhances visibility and makes all the targeted tissues clearly identifiable. Methylene blue is a valuable visual aid, ensuring a profound and radical debridement (Figure 1A,B). 

Once the medial parapatellar arthrotomy is performed, full access to the joint is achieved. Initial surgical maneuvers involve the meticulous removal of the medial and lateral synovial membrane, taking care to spare the quadriceps and patellar tendon laterally and the subcutaneous fat pad medially. All fibrotic tissue within the joint must be meticulously removed throughout the debridement process until the implants are free and well isolated. First, tissue samples must be sent for histological and microbiological exams: (i) medial and (ii) lateral synovial membrane. 

### 3.3. Step 3: Implant Removal, Channel Preparation, and Bone Loss Evaluation

Removing implants must be executed with care, considering the importance of minimizing bone loss. It is imperative to extract the prosthesis without causing excessive loss of bone from the tibial and femoral epiphysis and metaphysis. 

The implant removal follows a logical sequence that starts from the polyethylene liner, then the femoral component, and finally, the tibial component.

**Figure 2:**
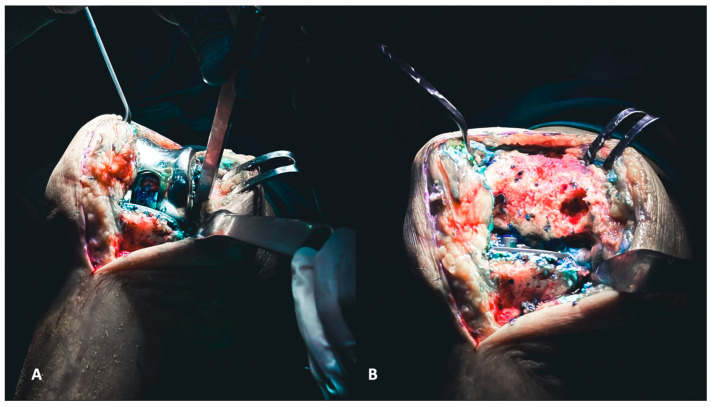
(**A**,**B**) The removal of the femoral component must be executed with the goal of preserving the entire bone stock for revision. Each surface of the femoral component needs to be carefully separated, both on the medial and lateral surfaces and the posterior surface (**A**), to ensure optimal preservation (**B**).

Depending on its morphology, the polyethylene liner can be lifted upwards using an osteotome placed under the liner or with the assistance of a Hohmann inside the liner. These maneuvers are often adequate for removing it from the tibial component. Subsequently, the femoral component can be removed. A reciprocating sawblade used to dissect the femur from the medial and lateral sides and an osteotome for the posterior chamfer and posterior condyle can ensure the extraction without creating cavitary defects in the condylar region (Figure 2A,B). The last component to be removed is the tibial component. The knee must be hyperflexed, and careful attention should be paid to the position of the tibial plateau, as the femoral condyles can often impinge and hinder the removal. 

**Figure 3:**
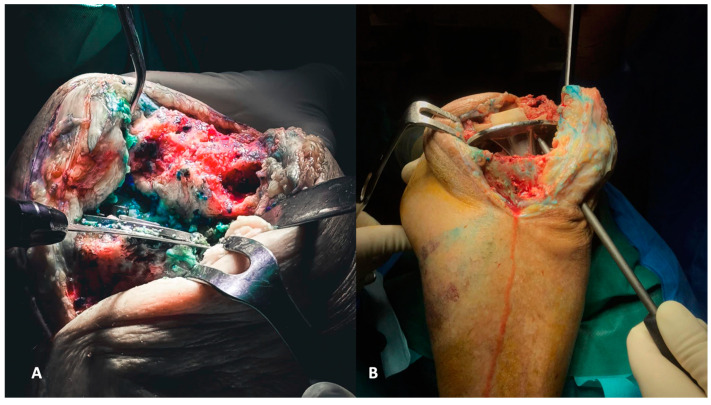
(**A**,**B**) The removal of tibial components, as for the femoral counterparts, requires careful execution to preserve the bone stock. The utilization of a reciprocating saw facilitates the separation of the component from the tibial plateau (**A**). Additionally, employing a pointed blunt impactor can aid in the removal process by hammering it from below (**B**).

A reciprocating saw blade dissects the tibial component (Figure 3A). Then a pointed blunt impactor is inserted through the metaphyseal tibial bone lateral to the patellar tendon to hammer the component (Figure 3B).

Once the implant is removed, the epiphyseal bone remains as the foundation for the subsequent reconstruction. A thorough debridement is essential to clean the bone from residual cement and other debris. Femoral and tibial channels must be meticulously cleaned using reamers with different diameters, debriding the intramedullary canal and promoting copious bleeding to enhance the healing process. Femoral and tibial periprosthetic tissue interfaces are obtained and sent for histological and microbiological examination. Because almost 30% of positive cultures in PJIs of the knees are recorded in the intramedullary canal [17], two samples from bone canals are routinely taken for microbiological analysis.

### 3.4. Step 4: Meta-Epiphyseal Reconstruction: Tibia and Femur

**Figure 4:**
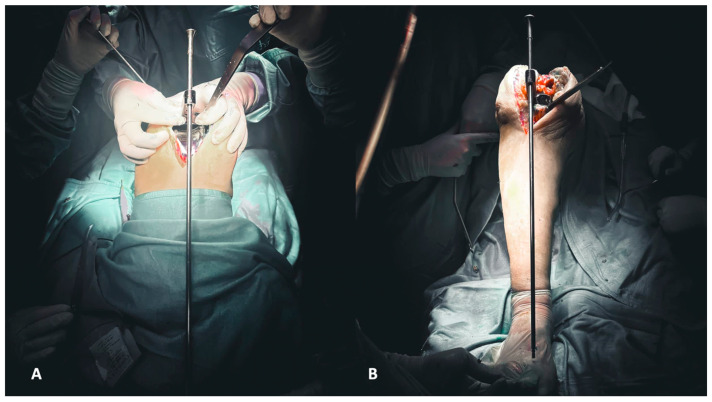
(**A**,**B**) Evaluation of the femoral (**A**) and tibial (**B**) axis through extra medullar methods.

**Figure 5:**
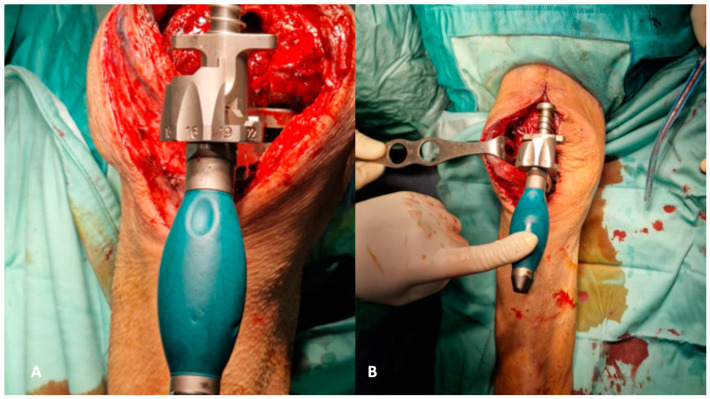
(**A**,**B**) Evaluation of flexion (**A**) and extension (**B**) gaps.

The first step of reconstruction is the evaluation of the tibial and femoral axis through extra medullar methods (Figure 4A,B) and the flexion and extension gaps (Figure 5A,B). Reconstruction starts at the tibia, affecting both flexion and extension gaps. 

**Figure 6:**
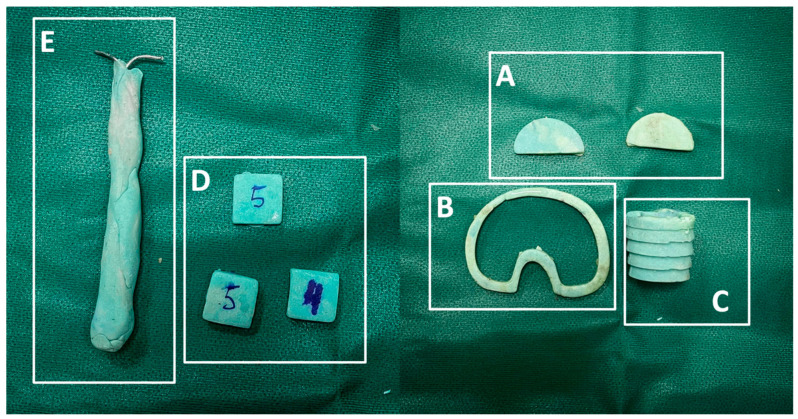
(**A**–**E**) Angular deformities can be addressed using hand-made antibiotic-loaded cement asymmetrical wedges applied to the medial or lateral tibial platform (**A**). When the tibial platform should be proximalized to fill the gap, a symmetric cemented augment should be prepared (**B**). To address metaphyseal bone defects, it is important to prepare and use a cemented filler shaped like a cone (**C**). Like the tibia, angular deformities on the femur’s coronal and traversal planes should be addressed using cemented augments to apply on distal or posterior femoral condyles (**D**) and self-made stem (rods, wires, or screws) to address the femoral and tibial canal (**E**).

At this stage, the surgeon evaluates the tibial epiphysis, identifying any varus/valgus deformities and bone defects, both segmental and cavitary. Angular deformities can be addressed using hand-made antibiotic-loaded cement asymmetrical wedges applied to the medial or lateral tibial platform (Figure 6A). It is crucial to verify the correct tibial axis multiple times during this procedure to plan a suitable correction. When the tibial platform should be proximalized to fill the gap, a symmetric cemented augment should be prepared (Figure 6B). To address metaphyseal bone defects, it is important to prepare and use a cemented filler shaped like a cone (Figure 6C), with sufficient space in the middle to accommodate the self-made stem (rods, wires, or screws) (Figure 6E) [18]. 

Like the tibia, angular deformities on the femur’s coronal and traversal planes should be addressed using cemented augments to apply on distal or posterior femoral condyles (Figure 6D). Symmetric distal and posterior augments should be used to fill extension and flexion gaps, respectively. If a cavitary bone defect is present, a cemented filler shaped like a cone (Figure 6B) must be positioned in the meta-diaphysis of the distal femur. Antibiotic-loaded bone cement with an extra dose of vancomycin (2 g of each 40 g of bone cement) and gentamycin (240 mg of each 40 g of bone cement) is used for augment and cone preparation [19].

Cement augments and cones, when handcrafted, may lack consistency in size and shape, thereby compromising reproducibility. To address this issue, certain companies provide products that enable surgeons to create precise augments and cones by molding bone cement into silicon molds. This ensures a reproducible technique and known sizing.

### 3.5. Step 5: Trial Component Positioning and Definitive Implantation

**Figure 7:**
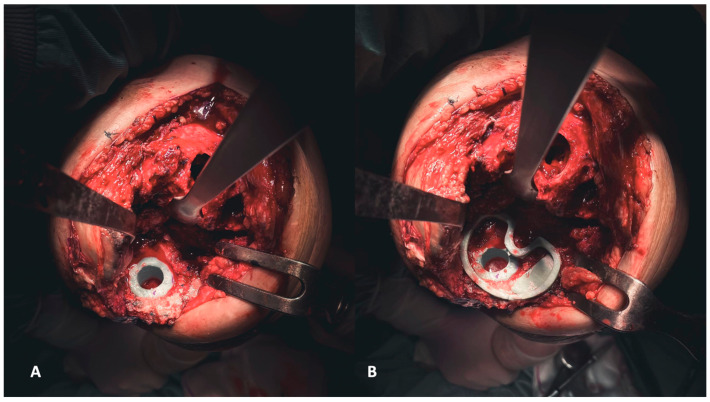
(**A**,**B**) Establishing the foundation involves addressing the tibial plateau. Initially, positioning the cone addresses metaphyseal cavitary defects (**A**), followed by the placement of symmetrical and asymmetrical augments both above (**B**).

**Figure 8:**
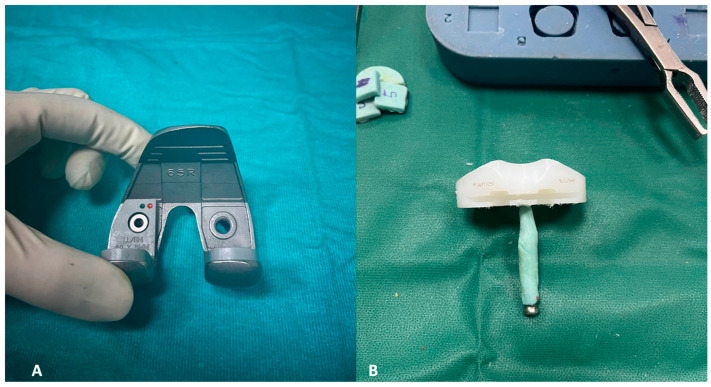
(**A**,**B**) The definitive components for the femur, accompanied by corresponding augments (**A**), and for the tibia. The latter involves a self-made stem created by inserting a screw into the polyethylene liner, subsequently covered in cement (**B**).

**Figure 9:**
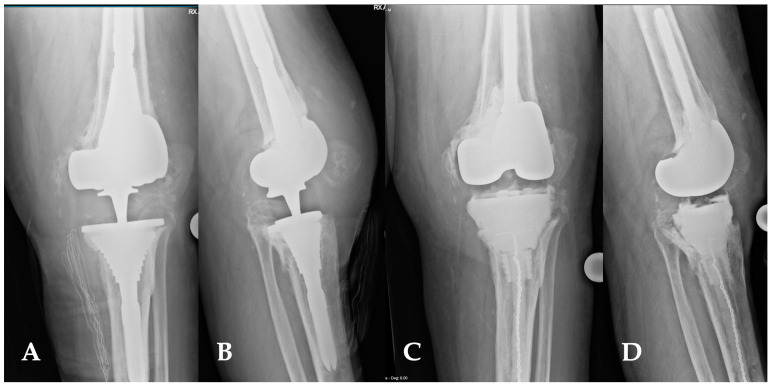
(**A**,**D**) This image depicts the comparison between preoperative and postoperative X-rays of a patient affected by periprosthetic joint infections of the knee (**A**,**B**). The patient’s characteristics met the inclusion criteria for the indication of a balanced articulating spacer. The spacer was constructed using cones and both asymmetrical and symmetrical augments, particularly to address the severe bone loss of the tibia (**C**,**D**).

A cruciate retaining (CR) femoral component trial is placed, and a check of correction of both rotational and axial deformities by using cemented augments with a predetermined thickness is performed. With the femoral trial component in position, the chosen ultra-congruent polyethylene liner is placed on the tibia with all the bone cement handmade components prepared before according to tibial bone defects. Then, we check the knee stability in flexion and extension, the range of motion, patella height and tracking, and knee varus/valgus axial deviation. Based on these findings, it is possible to move the joint line as desired proximally using bone cement handcrafted symmetrical augments on the tibial side, or more distally through bone cement handcrafted augments under both distal facets of the femoral component. Once the thickness and the size of the liner and the femoral component are chosen, the definitive components are opened. A fully threaded screw or fiche is inserted into the liner before being cemented on the tibial platform. Handcrafted cemented stems are prepared to attain sufficient antibiotic elution in the intramedullary canals of the femur and tibia. The tibial one is made through the cementation of the screw or fiche inserted in the liner, and the femur, instead, is created through a metal cerclage appropriately modeled and covered by cement. Both stems are placed through the self-made tibial and femoral cones. All the selected augments are set in position (Figure 7A,B), and the polyethylene liner and relative augments are first cemented on the tibial epiphysis. Once the liner is well fixed and the first cementation is solid, a second cementation is made, and the femoral CR component is cemented (Figure 8A,B and Figure 9A,B). 

### 3.6. Step 6: Antiseptic Irrigation Solution and Wound Closure

The irrigation solution used in the joint during the surgery also plays an important role. There are different possibilities, according to in-hospital availability and surgeons’ choice. In our technique, a solution containing Povidone-Iodine (Pl) at 0.3% dilution and Hydrogen Peroxide (H_2_O_2_) at 0.5% dilution is preferred in Gram-positive germ infections for three minutes of exposure once the femoral and tibial canals are properly closed; otherwise, in culture-negative infections or in the case of infections sustained by Gram-negative bacteria, a solution containing PI at 5% is used [20]. Primary wound closure is always preferred when possible. If not, an ortho-plastic approach is used through skin grafts or flaps, like the medial or lateral gastrocnemius flap [21].

## 4. Discussion

All cement spacers are still widely used worldwide to address PJIs in TKA in a two-stage approach [22]. Our concerns about this type of spacer are linked to the limitations well described in the current literature, such as the preformed sizes not allowing personalization to the patient, and the incapability to provide enough stability to the joint, strongly limiting the quality of life and knee function [9,23].

Moreover, the widespread use of 1.5-stage revision as a treatment in PJIs requires a stable and working knee; otherwise, the second stage is unavoidable. Thus, a metal-on-poly spacer is strongly suggested in these cases, allowing the surgeon to easily decide which treatment path to follow [24].

In fact, a metal-on-poly spacer enables patients to have earlier full weight bearing and quicker recovery of the knee’s range of motion, leading to a better quality of life after surgery [25]. Therefore, all these considerations lead us to apply this technique to almost every case of PJI in TKA, except when extensor mechanism disruption, severe bone loss, or mangled soft tissues are involved. In such cases, a static spacer is preferred [5].

While the surgical procedure duration may be slightly prolonged compared to using an unbalanced articulating spacer, the benefits of achieving proper spacer balance are fundamental for postoperative rehabilitation and physical therapy, which can be significantly enhanced and accelerated compared to conventional methods, without any sort of immobilization. A balanced and stable spacer facilitates earlier and more effective recovery of knee function compared to an unbalanced spacer. Consequently, despite potential minor time implications during surgery, the advantages gained in spacer balancing greatly outweigh any associated drawbacks and are crucial for optimizing patient outcomes. An improved quality of life and optimal knee function are intricately linked, exerting profound effects on patient health. These factors play a pivotal role in fostering comprehensive healing, encompassing not only infectious considerations but also psychological and physical dimensions. 

Furthermore, a meticulously crafted articulating spacer, balanced through precise cement augmentation of a predetermined size, empowers the surgeon to effectively plan for a potential second-stage revision. This foresight enables the anticipation of defects and the necessary augmentations [26]. Consequently, a streamlined surgical process not only minimizes operating time but also limits joint exposure, potentially reducing both intraoperative and postoperative complications. This is particularly crucial in the case of patients widely acknowledged as being at higher risk [27]. 

In terms of economic burden, the augmented articulating spacer carries a similar cost to other metal-on-poly spacers and is comparable in cost to most cement spacer instrumentation. However, it is important to note that while cement spacers may be less expensive, they are often inadequate in addressing many of the concerns outlined previously.

The limitations of this technique lie, clearly, in the handcrafted bone cement augments and cones, due to their lack of reproducibility and strong correlation with the surgeon’s skill in cement modeling. This can result in a poorer quality of the spacer and, consequently, an unstable and poorly balanced knee. Tools that assist surgeons in crafting the cement products, such as silicone stamps for cement molding, could be beneficial in addressing this issue.

## 5. Conclusions

This technique for metal-on-poly augmented spacers offers a more stable and balanced knee, thereby aiming to lead to an improved knee function and enhanced quality of life for patients undergoing two-stage revision in periprosthetic joint infections of total knee arthroplasty. Furthermore, it supports the surgeon in evaluating the 1.5-stage option, furnishing a trustworthy, secure, and stable working spacer. Additionally, it assists in strategic planning for the two-stage revision by delineating bone defects and specifying the required augmentations and cones.

## Data Availability

The data presented in this study are available on request from the corresponding author.
